# The 12 weeks, randomized, double-blinded, placebo-controlled human study to evaluate the effectiveness and safety of KGC deer antlers on the growth of children

**DOI:** 10.1097/MD.0000000000031567

**Published:** 2022-10-28

**Authors:** Sang Min Kim, Jin Yong Lee, Gyu Tae Chang, Su Min Hwangbo, Sun Haeng Lee

**Affiliations:** a Department of Korean Pediatrics, College of Korean Medicine Kyung Hee University, Kyung Hee University Medical Center, Dongdaemun-gu. Seoul, Republic of Korea; b Korea Institute of Korean Medicine, Yuseong-gu, Daejeon, Republic of Korea; c Department of Korean Pediatrics, College of Korean Medicine Kyung Hee University, Kyung Hee University Hospital at Gangdong, Gangdong-gu, Seoul, Republic of Korea; d Department of Clinical Korean Medicine, College of Korean Medicine, Kyung Hee University, Dongdaemun-gu, Seoul, Republic of Korea; e Department of Korean Pediatrics, College of Korean Medicine Kyung Hee University, Kyung Hee University Medical Center, Dongdaemun-gu, Seoul, Republic of Korea.

**Keywords:** body growth, deer antlers, height

## Abstract

Cervi parvum cornu is a dried section of the young horns of *Cervus nippon* Temminck, *Cervus elaphus* Linnaeus, or *Cervus canadensis* Erxleben. It is a representative yang-tonifying medicine that warms the viscera and bowels, activates the overall physiological function, and has effects such as tonifying kidney yang, replenishing essence and blood, and strengthening muscles and bones.

**Objective:**

This clinical study is aimed to evaluate the effectiveness of deer antler extract on child growth.

**Methods:**

This clinical trial is designed to be conducted on 100 children aged 3 to 12 years for 12 weeks (Trial registration code: KCT0007386). We will evaluate changes in height, height percentile, standard deviation score of height, weight, body mass index, waist circumference, hip circumference, bone age, predicted adult height estimated by bone age, human growth hormone level, insulin-like growth factor-1 (IGF-1) level, IGF-binding protein-3 (IGFBP-3) level, IGF-1/IGFBP-3 ratio, and estradiol level. Additionally, we also will evaluate the adverse events during the study.

## 1. Introduction

Growth is a typical feature of childhood and refers to a series of processes that result in body weight and height increasing with age. These processes are affected by heredity, chronic diseases, social environment, nutritional supply, and hormones.^[1]^

The United States US Food and Drug Administration has specified that the height of children with idiopathic short stature (ISS) is less than 2.25 standard deviations from the average of children of the same age and sex. Accordingly, if the predicted adult height is less than 160 cm for men and 150 cm for women, growth hormone (GH) treatment is necessary.^[2]^ Therapy with GH typically yields only modest gains in height (approximately 4–6 cm), and the height in adulthood would generally be below average despite therapy.^[3]^

A previous study proposed that the ISS should not be treated with GH (grade 2C) because there are considerable disadvantages regarding the cost and burden of long-term treatment.^[4]^ Further, there are wide inter-individual variations in treatment outcomes, including no increase in adult height for some children. Although the outcomes are not readily predictable, shorter parental height, older age, and shorter stature at the time of GH treatment initiation are correlated with lower efficacy. In addition, GH treatment becomes less effective as the treatment duration increases, and the amount of GH needs to be increased. If the treatment is stopped, the growth rate temporarily decreases further, and the safety from long-term complications has not been confirmed.^[5]^ According to a study conducted in Korea,^[6]^ domestic GH treatment costs about 10-14 million won per year; thus, cost-effectiveness must be considered, in addition to the fact that daily injections can cause psychological damage to children.^[7]^ Therefore, when GH treatment is considered, it is critical for the provider to discuss realistic expectations with the child and the family.

Considering that there is a social trend for achieving height growth following effective treatments,^[8]^ there is an increasing desire for conducting studies for evaluating the effectiveness of different materials and treatment methods for addressing growth problems. In this regard, materials derived from deer antlers are of great interest, as they are widely used for immunity enhancement, growth, and development of children.^[9]^ To date, studies have reported that deer antler extracts have anti-stress and anti-aging properties, as well as significant efficacy in improving various bodily functions such as hepatobiliary, cardiovascular, immune system, endocrine, hematopoietic action, and glucose metabolism..^[10]^ In this regard, there have been several experiments^[11–13]^ on cells or animals, which led to the assumption that the rapid growth caused by treatment with antler extracts will have a positive effect on the growth of children; however, studies evaluating the validity of this assumption on the human body are scarce.

Therefore, we have designed a prospective clinical trial study to investigate the effects of deer antler extracts on children growth. The results will elucidate the effects of KGC deer antler extracts on growth and the adverse events (AEs) possibly associated with the administration of KGC deer antler extracts in children.

## 2. Methods

### 2.1. Ethics approval

The study will be conducted in accordance with the principles of the Declaration of Helsinki, the Ethical Guidelines for Clinical Research, and the Institutional Review Board (IRB) of Kyung Hee University Korean Medicine Hospital (KOMCIRB 2021-08-003-006). The study protocol was registered with Clinical Research Information Service (KCT0007386). All modifications to the protocol will be approved by IRB before its execution (Additional file 1; http://links.lww.com/MD/H884). Korean medical doctors (researchers) will explain the study to the children and their guardians and acquire written informed consent. If adverse events occur during the study, appropriate medical treatment will be provided immediately until the participant recovers. All participants’ data will be documented by a code number, and the documents will be stored in locked file cabinets with controlled access. There is no plan or intention to use the results of this study for the individual recognition of functional raw materials for health functional foods.

### 2.2. Study design

This is a prospective double-group study protocol, which will be conducted on 100 children aged 3 to 12 years. This study is designed to observe the effectiveness and safety of KGC antlers for growth promotion. The study protocol conforms to the Standard Protocol Items: Recommendations for interventional trials.

### 2.3. Participants

**Figure 1.**
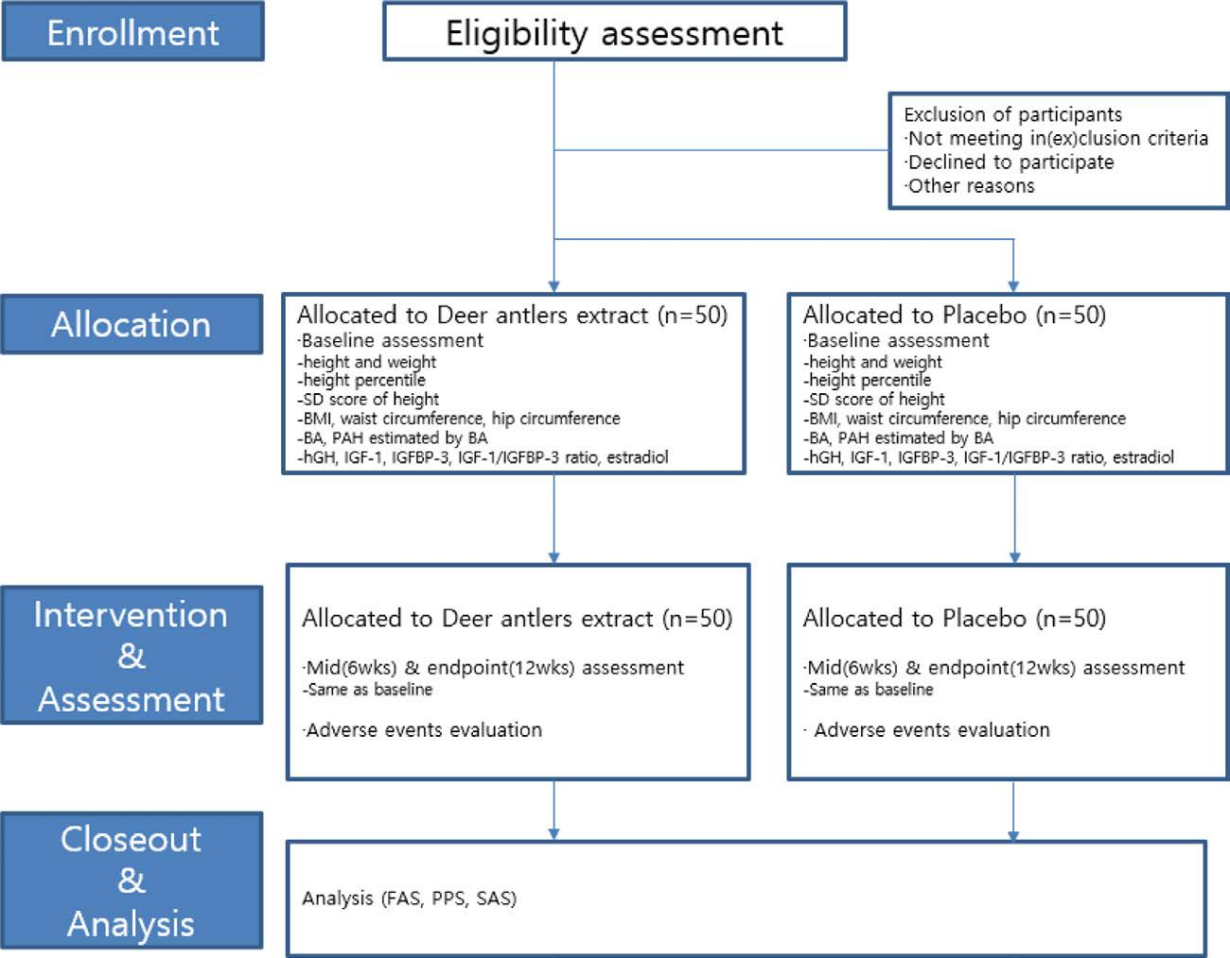
Schematic chart of the study process. BA = bone age, BMI = body mass index, FAS = full analysis set, hGH = human growth hormone, IGF-1 = insulin-like growth factor-1, IGFBP-3 = insulin-like growth factor binding protein-3, PAH = predicted adult height, PPS = per protocol set, SAS = safety assessment set, SD = standard deviation.

**Figure 2.**
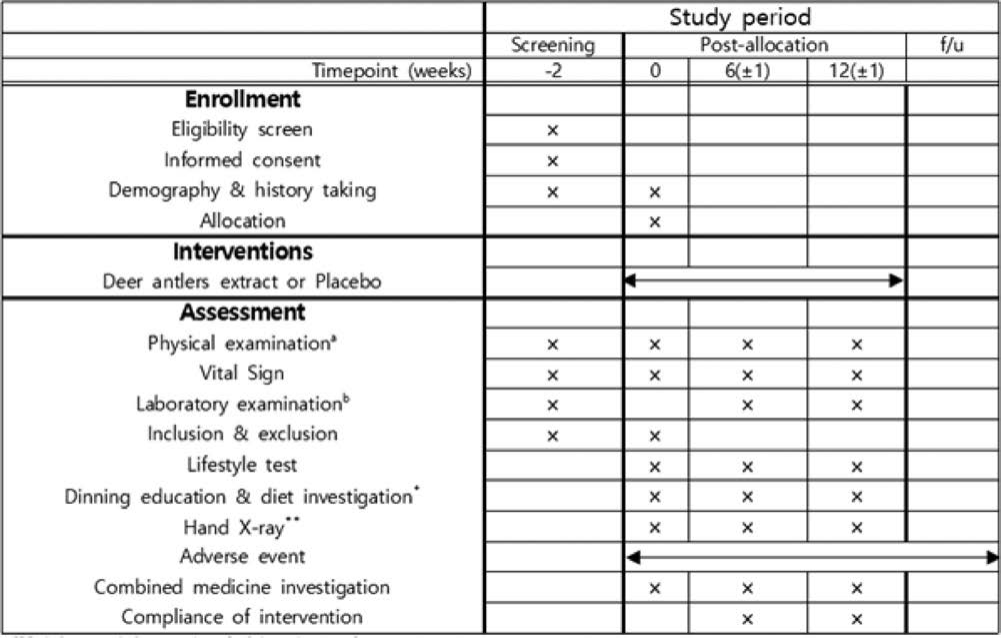
Schedule of enrollment, intervention, and assessments. (a) Height, weight, and waist and hip circumference. (b) Hematologic and Serum Chemistry test, Urine test, Hormone test, Estradiol. * Researchers will record the dining pattern of the day before using the 24-h recall method. Additionally, researchers will withdraw and check the dietary questionnaire handed out during the previous visit. ** If there was an examination within 4 weeks, it can be used.

One hundred children from the Kyung Hee University Medical Center will be recruited. The study will be advertised using posters and banners announcing the need for volunteer participants. The participants and their guardians will receive research information, such as objectives, procedures, and potential benefits and harms, through standardized dialogue. Informed consent will be obtained from the children and their guardians before the screening process. Participants are allowed to withdraw from the study at any time. The research procedure will be conducted as shown in Figures 1 and 2.

If it is determined that the number of study participants does not meet the expectations, changes or additions to the recruitment method will be implemented through discussion between the research sponsor, the person in charge, and the practitioner.

### 2.4. Sample size

The expected difference in height change was set to 0.45 cm.^[14]^ Based on this, the difference between groups was tripled and set at 1.35 cm. The standard deviation (σ) was set to 2.0, which is the standard for growth disorders (less than −2.0 standard deviation from the average of children of the same age and sex). Accordingly, the sample size was calculated using a 2-tailed 5% significance level, 80% power, and a 1:1 allocation. Accordingly, the required number of subjects per group is 35 and the total number of enrolled subjects is 100 (50 in each group), allowing for a 30% withdrawal rate.

### 2.5. Inclusion criteria

In this study all children (boys and girls) aged between 3 and 12 years, who agree to participate in this study and have written informed consent forms signed by their representatives, will be included.

### 2.6. Exclusion criteria

Children who have endocrine diseases (juvenile diabetes, hyperlipidemia, and hypertension), diseases causing growth retardation (growth hormone deficiency due to pituitary disease), chromosomal abnormalities, or abnormal appearances will not be included in the study. Additionally, children who have not been born with a normal range of weight (i.e., they were less than 2.6 kg or > 4.4 kg), have received hormone replacement therapy, calcitonin, phosphonate, and growth hormone treatment within 6 months prior to the screening, or have any clinically significant acute or chronic diseases of the cardiovascular, endocrine, immune, respiratory, hepatobiliary, kidney, urological, neuropsychiatric, musculoskeletal, inflammatory, haemato-oncological, or gastrointestinal system, are not considered for participation. In this regard, children who have taken medicines or health functional foods related to children’s height growth or antipsychotics within three months prior to the study, as well as who have participated in another clinical trial within 1 month prior to the screening test, will be excluded from the study. Furthermore, any child who is determined as inappropriate for study due to other reasons, such as abnormalities in clinical laboratory tests, will be excluded from the trial.

### 2.7. Intervention

The intervention group will receive deer antler extracts (1,86 mg/20 mL) for 12 weeks. All participants would be instructed to administer the extracts once a day before theirs meals. Further, the children in the control group will receive a placebo with the same frequency using the same method.

### 2.8. Random assignment

During subject enrollment, the investigator will assign a unique identification code to each subject in the order of enrollment. Participants who meet the inclusion criteria will be assigned a randomized number according to the randomization plan. The randomization table is a sequential application of the permutations of random numbers (random numbers of A or B) generated by the random number generation program of the safety assessment set system, starting from subject number 1. This was designed and generated in advance by an independent statistician using safety assessment set V9.4 before clinical research. As children and adolescents in the growth and development stages are targeted, they will be divided into four stratified groups and randomly assigned to each group (Table 1).

**Table 1:** Stratification of study subjects considering growth phase characteristics (gender and age)

Girls		Boys	
Group 1	Age between 3 and 9	Group 3	Age between 3 and 10
Group 2	Age between 9 and 12	Group 4	Age between 10 and 12

### 2.9. Outcome measurements

All data related to the measurements of outcome were directly entered into the an electronic case report file (e-CRF) by a clinical research coordinator who received appropriate training for clinical research.

#### 2.9..1. Primary outcome measurement.

Height will be measured using a portable stadiometer (Seca 213, Seca GmbH, Hamburg, Germany; measurement range: 20–205 cm) by a trained nurse at the same time of day with the participant barefoot, and the value will be rounded to 2 decimal places. Height and weight will be measured at baseline and at 6 and 12 weeks.

#### 2.9..2. Secondary *outcome measurement.*

##### 2.9..2..1. Weight and body mass index.

Weight will be measured while participants are in light clothing, using a load cell scale (CAS HE-30, Yangju, Republic of Korea; measuring range: 5–180 kg) by a trained nurse, and the value will be rounded to 2 decimal places. Body mass index will be calculated by dividing weight by the square of height.

##### 2.9..2..2. Percentile of height.

In this study, the height percentile will be measured at baseline and at 6 and 12 weeks. Accordingly, the recorded values will be interpreted using the growth chart of children and adolescents in Korea developed in 2017 to determine the growth status of participants.

##### 2.9..2..3. Waist and hip circumference.

Waist circumference and hip circumference will be measured three times (at baseline, and at 6 and 12 weeks) by the same researcher to prevent errors. Waist circumference will be measured in the middle between the lowest point of the ribs and the highest point of the pelvis according to the WHO standard. Hip circumference will be measured in a horizontal plane at the level of the maximum circumference of the hips and buttocks. Each value will be rounded to 2 decimal places.

##### 2.9..2..4. Bone age (BA) and predicted adult height (PAH).

BA and PAH will be measured at 6 and 12 weeks with VUNO Med BoneAge^TM^, an artificial intelligence program using the Greulich-Pyle (GP) method.

For unification of the analysis, for subjects whose PAH cannot be calculated with VUNO Med BoneAge^TM^ (chronologic age 4 years or older and bone age 7 years or less; bone age and chronological age are more than 2 years apart), PAH will be calculated using the Tanner–Whitehouse (TW) method. Furthermore, in case of 3 years old children for whom the PAH cannot be calculated even with the TW method, the predicted height will be calculated using a growth chart.

##### 2.9..2..5. Height standard deviation score (SDS) and height SDS for BA.

The height SDS will be calculated by subtracting the average height of the same age and sex from the measured height of the participants and dividing the value by the standard deviation of the height. Accordingly, the height SDS for bone age and the height SDS for chronological age will be calculated based on bone age and chronologic age, respectively.

##### 2.9..2..6. Insulin-like growth factor-1 (IGF-1), insulin-like growth factor-binding protein-3 (IGFBP-3), IGF-1/IGFBP-3 ratio, estradiol, and human growth hormone.

A total of four hormones including human growth hormone, IGF-1, IGFBP-3, and estradiol, are selected as indicators to determine the participants’ height growth status. Levels of these hormones will be measured via blood tests at baseline and 6 and 12 weeks to determine the amount of change after using antler extract. Blood collected for laboratory testing (human material) is collected and managed in the hospital, and is discarded immediately after analysis for clinical research. Secondary use does not apply.

### 2.10. Statistical analyses

In this study, analysis will be performed by defining the analysis group as safety, full analysis set (FAS), and per-protocol set analysis. The safety analysis group will be defined as all subjects who have been randomized and who have consumed at least 1 study food and will be evaluated for safety. The FAS analysis group refers to subjects for whom data on primary outcomes can be obtained among those who have taken research food at least once. The per protocol set analysis group includes subjects who have completed the study without serious violations (violation of inclusion and exclusion criteria, use of concomitant medications that are not permitted, and medication adherence less than 70%) according to the research protocol among subjects included in the FAS analysis group. If missing data occurs during FAS analysis or if a subject drops out before the clinical study is terminated, data analysis will be performed with the most recently obtained data as if it were obtained at that time (Last Observation Carried Forward Method).

Statistical analysis is intended to test whether there is a statistical difference between the test and control groups. Therefore, the mean, standard deviation, median, minimum, and maximum values of the outcome measurements will be calculated, and comparisons between the groups will be performed using a 2-sample t-test (Wilcoxon’s rank sum test when the assumption of normality distribution is not satisfied). For laboratory tests and vital sign results, a paired t-test will be used to analyze whether there will be a difference in the amount of change within the group at the end of administration of the test substance compared with before treatment (screening). Further, PAH will be sub-analyzed according to calculation methods such as the VunoMed, TW, and growth chart methods. Additionally, covariate analysis (analysis of covariance; ANCOVA) with stratification as a covariate and subgroup analysis for each stratification will be conducted.

### 2.11. Data monitoring

Data for this clinical study will be collected using e-CRF. Further, we will ensure that the data on the e-CRF conforms with the hand-written source document that and they are accurate, complete, readable, and timely. Only the researcher or a permitted person is permitted to edit the e-CRF and source documents.

The researchers should keep and secure the data and records related to the conduct of the clinical study in a safe place and preserve it for three years from the date of completion of the clinical study and the date of discontinuation. After completing the preparation of the result report, the clinical research-related documents should be handed over to the person in charge of storage. If the researcher intends to discard or move the clinical research-related records, the sponsor should be informed in advance.

Monitoring will be carried out to protect the rights and welfare of subjects; to verify the accuracy, completeness, and verifiability of data by comparing reported clinical research data with source documents; and to confirm whether clinical research is performed according to the approved protocol and management standards. Olive Healthcare Co. Ltd. (Seoul, Republic of Korea), a consignment institution for clinical research, monitors clinical research through regular institutional visits and phone calls, evaluates clinical research progress, and confirms whether researchers perform their duties according to research plans and regulations. During the institutional visit, the monitoring officer will check the archiving of original source documents, e-CRF, drug management records, and research-related data and discuss any discrepancies or problems with the clinical study records with the investigator.

### 2.12. Adverse events

An adverse event refers to all harmful and unintended signs (including signs and abnormalities in laboratory test results), symptoms, or diseases that occurred in subjects who have received the tested food. These adverse reactions do not necessarily have a causal relationship with the tested food and might include abnormal test results, clinically significant symptoms or signs, changes in physical examination results, hypersensitivity, and progression or worsening of existing diseases.

All serious adverse events occurring during the clinical study period, whether related to the tested food or not, should be reported to the sponsor within 24 hour of the investigator’s awareness, regardless of administration. Adverse reactions occurring up to 28 days after the last administration of the tested food, after the end of the clinical study, should be reported only if they are serious and related to the tested food.

If a suspected unexpected serious adverse reaction (SUSAR) has occurred, the principal investigator should report it to the IRB, to decide whether the study should be continued or discontinued, and report it to the Minister of Food and Drug Safety promptly within the period specified in each of the following items through the sponsor:

- SUSAR that causes death or threatens life must be reported to the Minister of Food and Drug Safety as soon as possible through telephone, fax, or documents within seven days of the first report or knowledge of the sponsor, and a complete report additional reports must be made within 15 days of the date of first knowledge.
- All other SUSAR should be reported as soon as possible within 15 days from the date the sponsor receives or becomes aware of the fact.

### 2.13. Concomitant drugs and treatments

#### 2.13..1. Acceptable drugs or treatments.

The drugs listed below are approved for concomitant use at the discretion of the investigator.

- Drugs that have been regularly administered within 4 weeks prior to visit 1
- It is considered that it will not affect the interpretation of the results of this clinical study among the concomitant drugs that the subject has been taking before participating in this clinical study.
- Drugs used temporarily for the treatment of other diseases or adverse reactions

When administering all concomitant drugs, information about the drug (product name, administration purpose, administration dose, administration period) should be recorded in detail in the subject’s e-CRF.

#### 2.13..2. Prohibited drugs or treatments.

- Administration of growth hormone drugs and injections
- Food oriental medicine related to height growth food and growth promotion
- The case of administering other foods and drugs is defined as a treatment change, which can be determined by the attending physician. However, temporarily increasing the dose of the currently administered drug is not a reason for a change in treatment.

## 3. Discussion

With the development of science and technology, the number of children with growth difficulties due to hunger has decreased in many developed countries.^[15]^ According to a survey,^[16]^ the ratio of households to ensure food safety (i.e., the proportion of those who answered that the whole family ate a sufficient amount and type of food as desired) has shown a gradual increase over the past 10 years, confirming that the quality of nutritional intake has improved compared to the previous status. However, for the majority of parents, the growth of their children is a major concern, and parents are putting a lot of effort and investment into achieving better growth outcomes. According to a survey on the use of Korean medicine and Korean herbal medicine consumption in Korea in 2020,^[17]^ the interest in growth is high enough that 24.4% of children under the age of 19 used Korean medicine for the purpose of growth.

In practice, short stature is defined as 2 standard deviations below the average height of children of the same sex and age. However, even children who fall within the normal range desire to have larger heights, so they often visit medical institutions or find various ways to promote growth.^[18]^ Further, the average height continues to increase due to the improvements in eating habits and quality of life. However, a psychological burden might be imposed and persist into adulthood if growth does not happen smoothly according to the social atmosphere that favors taller heights.

Research on materials that can promote growth in such atmospheres is continuously being conducted. In particular, several experimental studies have been conducted on the growth effect of herbal medicines on bone growth, including single drugs such as astragalus and deer antlers and complex drugs containing Poria cocos or Dioscorea Rhizoma.^[19]^

Deer antlers are cut and dried young male antlers of Cervus nippon Temminck (梅花鹿), Cervus elaphus Linne (馬鹿), or Cervus canadensis Erxleben (大鹿) and are the fastest growing part of the animal tissues. Insulin-like growth factor-1 (IGF-1), which promotes bone growth, is produced in growing deer antlers, and IGF-1 is known to play an important role in the metabolism of osteoblasts that grow bones. Deer antler is originally a representative tonifying yang medicine (補陽藥) and has effects such as tonifying kidney yang (補腎阳), replenishing essence and blood (益精血), and strengthening muscle and bone (强筋骨).^[20]^ Kidney yang (腎阳) activates overall physiological function by warming the organs of the human body. Based on the efficacy of antlers, this clinical study is designed to prove the safety and effectiveness of promoting height and growth in children and adolescents.

The primary outcome item, height change, is the most intuitive item for judging a child’s growth. Generally, children older than 2 years whose height velocity is less than 4 cm/year should be monitored carefully for chronic nutritional deficits or other causes of short stature because at least 95% of children grow faster than 4 cm/year.^[21]^ However, since each subject’s current growth status is different, items such as SDS and height percentile are selected as secondary outcome measurements. In addition, since length growth is not the only growth indicator, weight growth-related indicators such as weight, waist circumference, and hip circumference will be also measured. Further, to determine the effect on future growth, bone age and predicted adult height will be used for growth prediction.

Among the secondary outcome items, hormone-related items including IGF-1, IGFBP-3, and estradiol levels will be also evaluated. IGF and IGFBP-3 are major indicators for measuring the effect of growth hormones on peripheral tissues and are frequently used indicators in studies confirming the effect of growth. IGF-1 is a hormone that functions as a major mediator of GH-stimulated somatic growth, as well as a mediator of GH-independent anabolic responses in many cells and tissues.^[22]^ IGF-1 is synthesized in the liver and is secreted into the blood under the control of GH. Further, a major function of IGFBP is to transport IGF, which controls access to the extravascular space, as well as tissue localization and distribution. IGFBP-3 is the most abundant form in plasma, with the highest affinity for IGF-1, and is in a saturated state.^[23]^ Estradiol is an estrogen steroid hormone and a major female sex hormone. Additionally, it has important effects on many other tissues, including fat, skin, liver, and the brain, especially estradiol, which has a profound effect on bone. Further, lack of estradiol would result in delayed epiphyseal.^[24]^ By examining the level of estradiol, we also wanted to confirm that antler extract does not interfere with female hormone levels and causes precocious puberty or other hormone-related symptoms.

A limitation of this study is that the stratification will proceed after receiving the study participants without considering the number of participants by age or gender. Further, as the study progresses, the age or sex might influence and bias the results. However, this conforms with the aim of this study, which is to examine the growth effect of antler extract for all children aged 3 to 12 years. Accordingly, additional research should be conducted to compare the growth effects according to age or sex.

In addition, it can be considered that food intake, which should be considered in many growth-related studies, will also affect the growth effect of deer antler extract. Since it was essentially impossible to control the diet and amount of food of the study participants, to reduce the deviation as much as possible, the test was conducted with a lifestyle test paper for children and adolescents, and a dietary questionnaire was distributed to record food intake while eating the study food. For a more precise study, it is necessary in the future to conduct a more detailed stratification classification, by considering the diet and meal amount based on the recorded data, and compare the results before and after taking the test drug in each stratified group.

The human body test protocol and case record file for this study were developed through the approval process several times under the management of the Institutional Review Board of Kyung Hee University Korean Medicine Hospital. Further, additional revision and supplementation processes might be performed for various reasons that might occur during the human application test. It is expected that significant results for the efficacy and safety of antler extract for pediatric height growth can be obtained through human application studies using this protocol.

## Acknowledgments

The authors thank Editage (www.editage.co.kr) for English language editing.

## Author contribution

**Conceptualization:** Sun Haeng Lee.

**Date curation:** Sang Min Kim.

**Funding acquisition:** Jin Yong Lee.

**Investigation:** Su Min Hwangbo.

**Methodology:** Gyu Tae Chang.

**Project administration:** Sun Haeng Lee.

**Visualization:** Sang Min Kim.

**Writing – original draft:** Sang Min Kim.

**Writing – review & editing:** Sun Haeng Lee, Jin Yong Lee, Gyu Tae Chang, Su Min Hwangbo.

## Supplementary Material


