# Supplementary material for: The 12 weeks, randomized, double-blinded, placebo-controlled human study to evaluate the effectiveness and safety of KGC deer antlers on the growth of children
Source: Medicine (Baltimore). 2022 Oct 28;101(43):e28397. doi: 10.1097/MD.0000000000031567 (PMC9622584; doi:10.1097/MD.0000000000031567)
Supplement: Supplementary file 1 [file medi-101-e28397-s001.pdf]

Additional file 1. Protocol version

Version	Date	Action
1.0	2021-May-06	Protocol draft development
2.0	2021-Jun-25	Outcome measurement revision
		Add participant stratified allocation
3.0	2021-Jun-30	Add secondary outcome measurement & assessment methods of bone age
3.1	2021-Aug-20	Add of participants recruitment method
3.2	2021-Aug-23	Add management and disposal of human material, participants support, data safety monitoring description
3.3	2021-Sep-13	Revision of the number of subjects and the basis for calculation
3.4	2021-Oct-06	Change the purpose of the test, Statistical analysis method added to stratified assignment
3.5	2021-Nov-08	Add the main ingredients of raw materials, basis for capacity setting
		Term change: Health functional food → Food
3.6	2021-Dec-14	Term change: Human application study → Clinical trial
3.7	2022-May-03	Add lab test items - Estradiol
3.8	2022-Jun-13	Add details related to bone age and predicted adult height
